# Xylose Metabolism and Transport in *Bacillus subtilis* and Its Application to D-Ribose Production

**DOI:** 10.4014/jmb.2504.04021

**Published:** 2025-04-25

**Authors:** Yong-Cheol Park

**Affiliations:** Department of Bio and Fermentation Convergence Technology, Kookmin University, Seoul 02707, Republic of Korea

**Keywords:** Xylose, D-ribose, *Bacillus subtilis*, xylose operon, xylose transport, transketolase mutant

## Abstract

Xylose is a five-carbon sugar and the second abundant mono-saccharide in lignocellulosic biomass. Xylose is not only a sugar substitute by itself, but also a good carbon source for the microbial and enzymatic synthesis of various valuable biomaterials. Most microorganisms are able to uptake and consume xylose as a sole carbon source because they possess specific transport systems and metabolic enzymes. *Bacillus subtilis* is a representative Gram-positive bacterium commercially used for enzyme and food production. Even though *B. subtilis* is popular in genetic and protein engineering, its application for metabolic engineering has been limited. Meanwhile, D-ribose is a five-carbon sugar and essential component in nucleotides, ATP, NAD, coenzyme A and so on. It boosts healthy effects on the human body such as enhancement of muscle performance and tolerance to myocardial ischemia. To produce D-ribose from xylose in *B. subtilis*, a comprehensive review on xylose metabolic regulation, xylose transport, and D-ribose biosynthetic engineering and fermentation process was provided. It would be useful for production of other valuable metabolites from xylose in *B. subtilis*.

## Introduction

Xylose present in lignocellulosic biomass is the second abundant mono-sugar in nature. As the substrate for valuable materials production, it is widely used in foods, cosmetics, human health and chemical industries. Also, xylose is able to be used as a carbon source for microbial cell growth and metabolite production. *Bacillus* is a rod-type Gram positive bacteria and found in most ecosystems. With high ability of protein production and secretion, *Bacillus* has been engineered and developed for manufacturing commercial enzymes such as amylase, lipase and protease. *Bacillus* has a big potential as a host strain for foreign gene expression, but metabolic engineering of *Bacillus* strains for valuable biomaterials production from xylose has been limited because of insufficient information of xylose metabolism in *Bacillus*. Meanwhile, D-ribose is a representative five carbon sugar in the human body and is a component in important metabolites such as ATP, NAD, DNA, RNA and so on [1]. As a commercial supplement of health-boosting effect, D-ribose is synthesized by chemical, enzymatic and microbial processes. Especially, transketolase-deficient *Bacillus* strains are known to be able to produce D-ribose by fermentation processes [2].

For understanding the xylose metabolism in *Bacillus subtilis* and its application for D-ribose, this review focused on (1) the regulation of the xylose metabolism including carbon catabolite repression and xylose operon, (2) the xylose transport system with general transport systems and *Bacillus*-specific xylose transport system and (3) production of D-ribose harboring its properties, metabolic pathway and production from xylose and other sugars.

## Regulation of Xylose Metabolism in *B. subtilis*

### General Carbon Catabolite Repression

*Bacillus subtilis* is a well-known Gram positive bacterium and has been chosen as a microbial host for commercial production of proteins and chemicals [3]. *B. subtilis* is able to consume a series of mono and di-saccharides since it has the genes coding for their corresponding metabolic enzymes. Enteric bacteria such as *Escherichia coli* can metabolize more than hundred different carbohydrates but *B. subtilis* can use fewer than 20 carbohydrates (mono-or disaccharides) as single carbon source [4]. Expression of genes involved in sugar metabolism depends on the presence or absence of carbon sources. Sugar metabolism was regulated by three control mechanisms at the transcription level: (1) repression of gene transcription by its repressor protein or induction by binding with an inducer, (2) carbon catabolite repression (CCR) mediated by a *trans*-acting global regulator and (3) inducer exclusion coupled with the phosphoenolpyruvate:sugar transferring system (PTS).

**Fig. 1:**
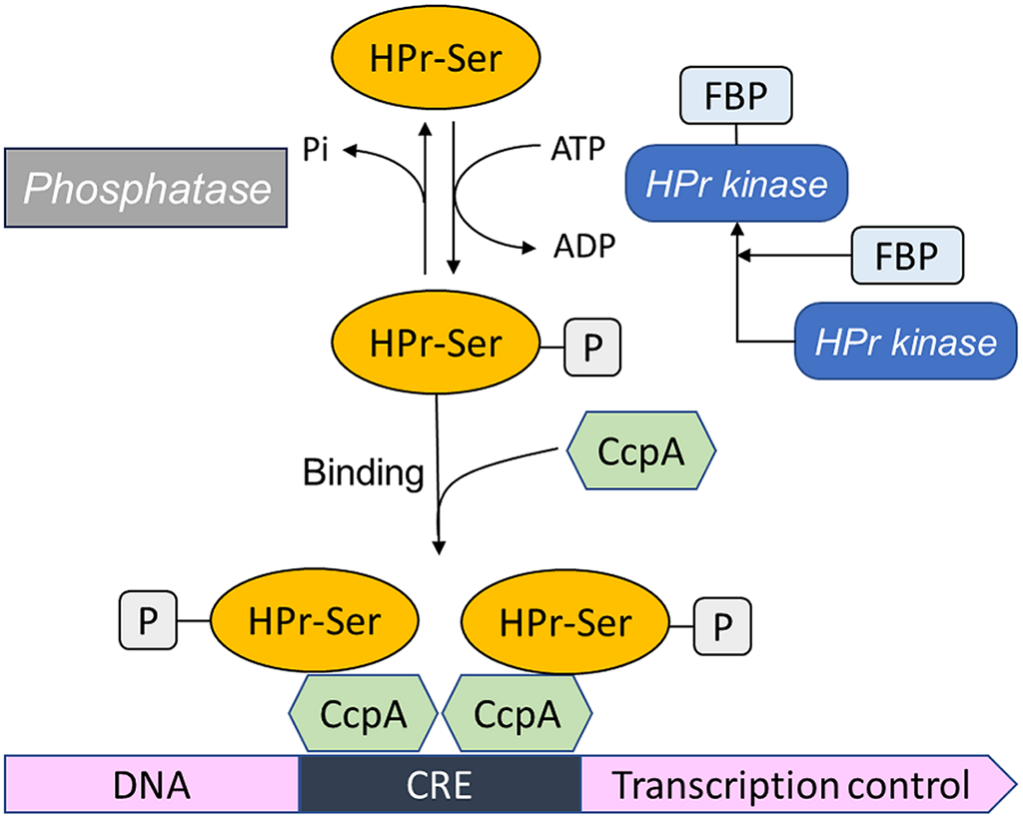
Model of the carbon catabolite repression mediated by HPr and CcpA in *B. subtilis*. Metabolites and proteins abbreviated as follows; FBP, fructose-1,6-bisphosphate; HPr-P, HPr protein phosphorylated at serine-46 residue; CcpA, carbon catabolite repression protein A; CRE, catabolite responsive element.

In the general CCR mechanism of *Bacillus* species (Fig. 1), the intracellular sensors of sugar availability are PTS and HPr kinase. HPr protein, an element of PTS can be phosphorylated at two distinct sites in contrast to the case of *E. coli*. A histidyl residue (His-15) is the target of phosphoenolpyruvate- and EI-dependent phosphorylation, whereas a seryl residue (Ser-46) is phosphorylated by metabolite-activated ATP-dependent kinase (HPr kinase)[5]. HPr phosphorylated at Ser-46 residue (HPr-Ser46-P) acts as a CCR regulator when binding with a global regulator. HPr kinase possessed bi-functional activities, showing both phosphorylation and dephosphorylation of HPr protein at Ser-46 according to the nutrient status of the cell [5]. Phosphorylation activity of HPr kinase is allosterically activated by elevated level of fructose-1,6-bisphosphate and inhibited by inorganic phosphate. In contrast, HPr phosphatase activity is stimulated by inorganic phosphate [6]. Crh protein can also be phosphorylated by HprK kinase and is involved in CCR [6]. Crh exhibited high sequence identity (45%) to the histidine-containing protein HPr but contains no catalytic His-15, the site of PEP-dependent phosphorylation in HPr [7]. A CcpA binding site of Crh protein phosphorylated at the seryl residue was proposed on the basis of highly conserved surface side-chains between Crh and HPr.

Unlike *E. coli*, cyclic AMP was not detected in low-GC content Gram-positive bacteria. Negative regulation mechanism of *B. subtilis* CCR derived by a *trans*-acting global regulator is completely different from the positive regulation operative in enteric bacteria by cyclic AMP and its receptor protein (CAP). Carbon catabolite repression protein A (CcpA, BG10376), a member of the GalR/LacI family of bacterial regulator protein possesses 36.8 kDa molecular weight, pI 5.06 and a highly conserved helix-turn-helix DNA-binding motif [8]. CcpA present in several bacilli and other Gram-positive bacteria binds with a cis-acting nucleotide, catabolite-responsive element (CRE) in the promoter or gene-coding region. CcpA consists of the N-terminal DNA-binding domain and the C-terminal protein core interacting allosterically with HPr-Ser46-P [5]. The affinity of CcpA for CRE sites of the *amyE* gene, *gnt* operon and sugar utilization operon increased in the presence of HPr-Ser46-P [5, 9, 10]. The CcpA/ HPr-Ser46-P complex is attached to the CRE region as a dimmer in a tetrameric protein complex [11]. CcpA protein not only regulates the transcription of the genes involved in most carbon metabolisms but also acts as a positive regulator of carbon excretion pathways. CcpA triggered the transcription of the acetoin biosynthesis operon (*alsSD*) and the acetate excretion genes (*ackA/pta*) in addition of glucose [12]. CcpA mutant strains exhibited elimination of catabolite repression but a severe growth defect on minimal media containing glucose, indicating that CcpA was necessary for full expression of glycolytic enzymes and for efficient ammonium assimilation [13]. High throughput technologies such as DNA microarray and proteomic analysis allowed the definition of a broad spectrum of novel regulation process mediated by CcpA [14]. Numerous catabolic/anabolic genes and operons in *B. subtilis* contained the CRE sequences. Mutagenesis studies or footprint and methylation interference experiments elucidated the CRE sequences in *B. subtilis*: the a–amylase gene (*amyE*) [9], the xylose (*xyl*) operon [15], the acetyl CoA synthetase (*acsA*) gene and acetoin catabolic (*acu*) operon [16]. Analysis of the CRE sequences of *B. subtilis* *amyE* and *acsA* genes and the *xyl* operon exhibited the palindromic sequence of the consensus TGWAARCG TTWNCW.

### Xylose Operon

**Fig. 2:**
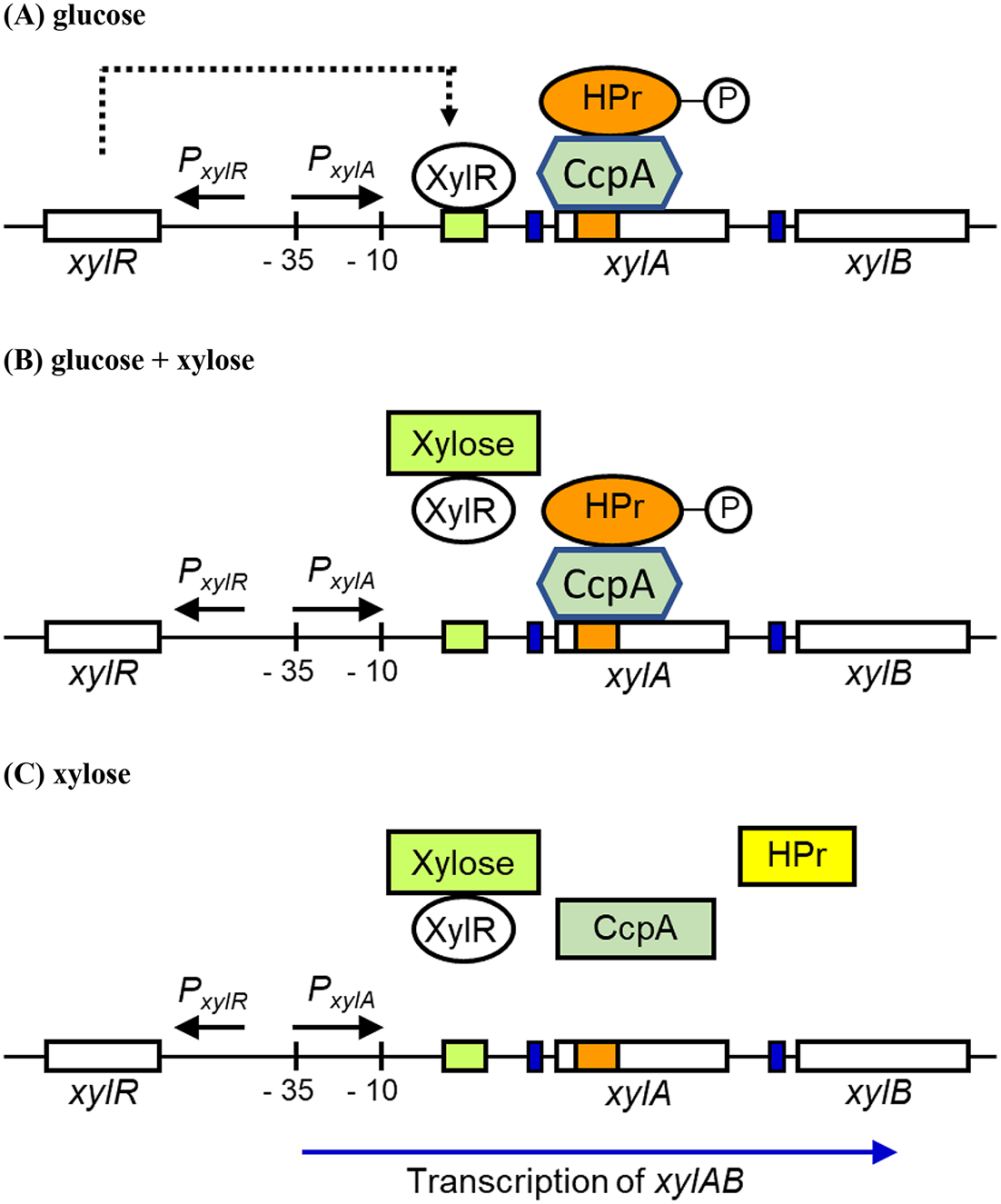
Mechanism of the xylose operon in *B. subtilis*. The *xylA*, *xylB* and *xylR* genes encoded xylose isomerase, xylulokinase and *xyl* repressor (XylR). Black box presented the catabolite responsive element (CRE) and slashed one indicated the XylR binding site. Gray box showed the ribosome binding site. HPr-P and CcpA abbreviated HPr protein phosphorylated at serine 46 residue and carbon catabolite repression protein A, respectively. (**A**) In the presence of glucose, transcription of the *xylA* and *xylB* genes does not occur since XylR and CcpA/HPr-P complex bind with XylR binding site and CRE, respectively. (**B**) In the presence of glucose and xylose, CcpA/HPr-P complex prevents the transcription of the *xylAB* genes in spite of XylR elimination by xylose addition. (**C**) In the absence of glucose and presence of xylose, decomposition of CcpA/HPr-P complex and elimination of XylR protein by xylose addition allow the expression of the *xylA* and *xylB* genes.

Bacteria able to use xylose inherently possess the xylose metabolizing enzymes such as xylose isomerase (EC 5.3.1.5) and xylulokinase (EC 2.7.1.17). Genes involved in xylose metabolism are mostly assembled into the xylose operon which contains one promoter controlling its genes expression. Firstly, a fragment of *B. subtilis* DNA coding for xylose isomerase and xylulokinase was isolated from a *BamHI* digestion pool [17]. Sequence analysis of upstream from the translational initiation codon showed that ATG was preceded by a Shine-Dalgano sequence (AAGGAG) locating at -14 position. There is a typical promoter structure for *B. subtilis* containing an upstream poly (A) block with 14 As followed by two hexamers, -10 sequence (TAAGAT) and -35 sequence (TTGAAA) spaced by 17 bp [18]. The xylulokinase gene (*xylB*) is located to the right of the xylose isomerase gene (*xylA*) and its transcription is subject to the xylose isomerase promoter [17]. Meanwhile, roles and actions of the catabolite repression factors on the xylose operon in the presence of glucose or/and xylose were described in Fig. 2. The regulation mechanism of the *B. subtilis* *xyl* operon was intensively investigated with the *lacZ* fusion system under the *xylA* promoter. When the expression level of β–galactosidase induced by xylose alone was 100-fold, the expression of the indicator gene in the presence of glucose and xylose or the absence of xylose was repressed to 30% of the fully induced level, indicating that repression was mediated in a *trans* mode by a negative regulator, XylR which was expressed at a low level in *B. subtilis* [18]. *In vitro* characterization of the *xyl* operator bound with XylR and competition assays using synthetic *xyl* operators revealed that the *xyl* operator consisted of ten palindromic base pairs flanking five central non-palindromic base pairs (TTAGTTTGTTTGGGCAACAAACTAA) [19]. Induction studies with several five carbon sugars suggested that xylose was the only molecular inducer of the xylose operon. Expression of the xylose operon was also regulated by CCR (glucose repression). A deletion of *xylR* encoding the Xyl repressor did not affect glucose repression [20]. The *cis* element mediating glucose repression was identified to be a 34 bp segment located at position +125 downstream of the *xylA* promoter in the translated region of the xylose isomerase gene, which had homology to various proposed consensus sequences for catabolite repression in *B. subtilis* [20]. Repression response studies were carried out by the modulation of XylR and CRE. Glucose, fructose and glycerol triggered the CRE-mediated response, whereas only glucose triggered the XylR-mediated response in addition to the CRE-dependent route [21]. Deletion of CRE was more efficient to overcome the transcriptional inhibition of the *xylA* promoter in the presence of glucose and xylose than deletion of the *xylR* gene [22]. A double mutant of XylR and CRE restored the expression level of *lacZ* under the *xylA* promoter. In *B. subtilis*, CcpA and HPr-Ser46-P play a central role in carbon catabolite repression as described earlier. CcpA binding with HPr-Ser46-P prevents the expression of the xylose operon by its attachment onto the CRE site. Deletion of *ccpA* in *B. subtilis* and *B. megaterium* led to not only the loss of CCR but also a markedly decreased growth rate, which seemed to be attributed to the reduction of the glycolysis efficiency [13].

## Xylose Transport System in *B. subtilis*

### General Sugar Transport Systems

**Fig. 3:**
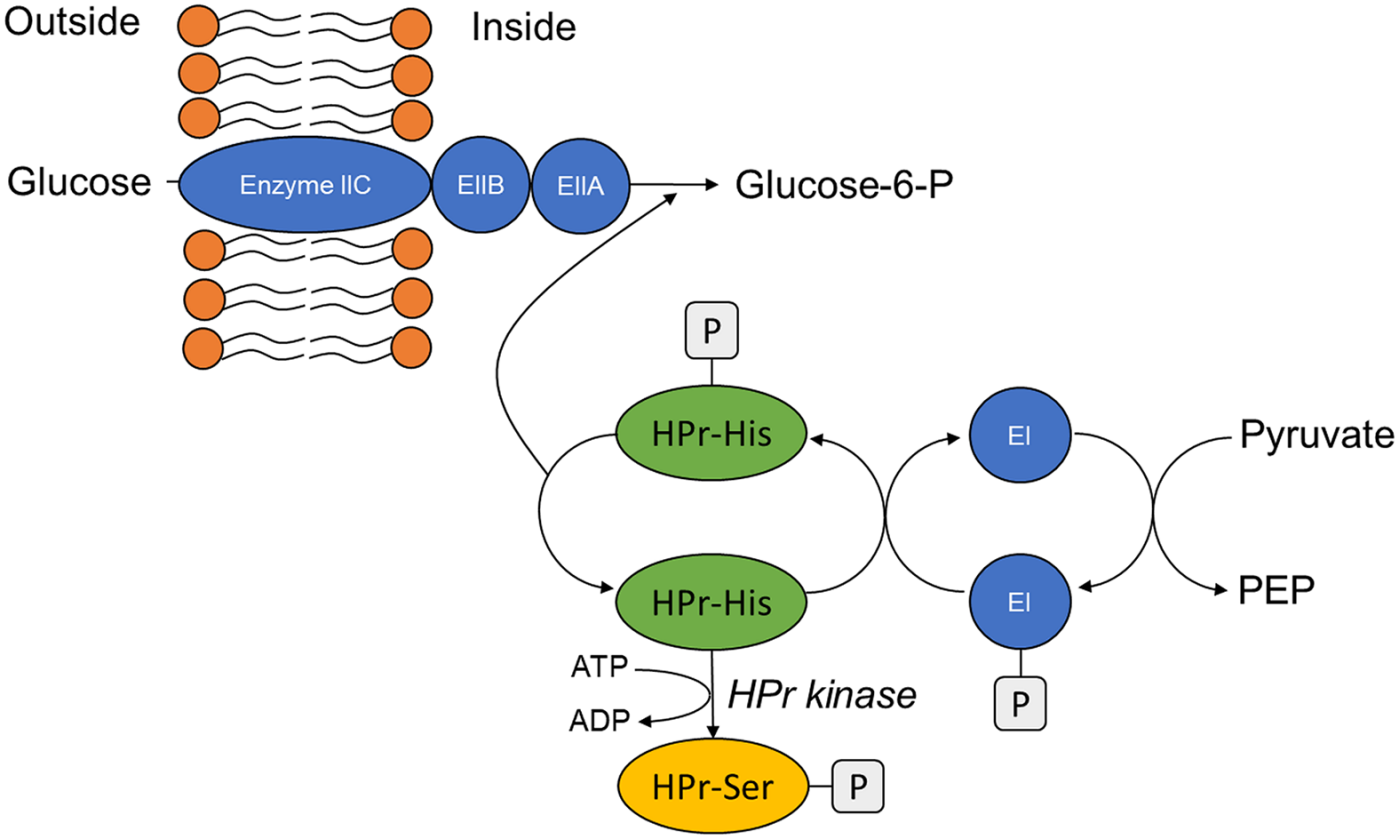
Phosphoenolpyruvate:sugar transferring system (PTS) of *B. subtilis*. PTS elements abbreviated as follows; PEP, phosphoenolpyruvate; EI, enzyme I; EI-P, enzyme I containing a phosphorylated histidyl residue; HPr-His-P, HPr protein phosphorylated at histidine-15 residue; HPr-Ser-P, HPr protein phosphorylated at serine-46 residue; EIIABC, enzyme IIABC

Cytoplasmic membrane transporters consist of one or more protein components, but typically they include at least one protein that spans the membrane with multiple transmembrane a–helical segments. Sugar transport proteins in *B. subtilis* fall into four classes of transporter types: channel proteins, secondary active transporters, primary active transporters and group translocators accompanied with phosphorylation of sugar [23]. At first, the channel type transporter catalyzes facilitated diffusion by an energy-independent process and by passage through an aqueous pore or channel in transmembrane. A representative channel transporter is GlpF protein to take up glycerol, of which gene is involved in the *glpFK* operon encoding glycerol kinase (GlpK) additionally [24]. Secondly, many secondary active transporters use the proton motive force or the sodium motive force as a driving force to transport solutes against a concentration gradient in the presence of a membrane potential that is negative inside. This category can be divided into three types of transport: symporter, antiporter and uniporter. Amino acids, peptides, simple sugars, oligosaccharides, organic phosphate esters, organic acids and vitamins were transported by this type of transporters. GlcP for glucose/mannose:H^+^ symport, and AraE protein for L-arabinose uptake, GntP for gluconate, LctP for lactate and GlpT for glycerol-3-phosphate belong to the secondary transport system using the motive force [23, 25-27]. Thirdly, primary active transporters indicate the ATP-binding cassette (ABC) type of delivery machines. These transporters consist of two transmembrane proteins, one or two peripheral-membrane ATP-binding protein(s) localized on the cytoplasmic side of the membrane, and a high-affinity solute-binding protein [23]. The ribose operon in *B. subtilis* contained RbsA protein displaying the characteristic of the ABC group of transporters [28]. Finally, group translocation is mediated by the phosphoenolpyruvate:sugar transferring system (PTS). It is composed of the general energy coupling proteins, enzyme I (EI) and HPr protein encoded by the *ptsI* and *ptsH* genes, respectively, which are shared by most PTS sugars, and the sugar specific enzyme II (EII). Delivery mechanism of the glucose specific PTS was described in Fig. 3. Phosphoenolpyruvate phosphorylates EI initially. Phosphohistidyl-EI transfers the phosphoryl group to the histidine residue 15 of HPr protein. This phosphoryl group is delivered to the enzyme IIA and IIB in their orders. Finally, enzyme IIC transfers the phosphoryl group to glucose and glucose-6-phosphate is transported into *B. subtilis* cells. Fifteen PTS permeases were found by the genome analysis of *B. subtilis*. Glucose, a-glucoside, lactose, mannose and fructose/mannitol families of PTS permeases were identified [29].

### Xylose Transport in Gram-Positive Bacteria Including *B. subtilis*

Several Gram-positive bacteria can take up D-xylose by their specific D-xylose transport systems. Batch culture of *B. megaterium* WH331 in a minimal medium with a D-glucose and D-xylose mixture exhibited the diauxic growth and the rapid consumption of D-xylose after glucose depletion. D-Xylose:H^+^ symporter encoded by the *xylT* gene is the last gene in the *xyl* operon of *B. megaterium* and its amino acid sequence showed 54% of identity to the XylE of *E. coli* [30]. Kinetic analysis of D-xylose uptake indicated a single D-xylose-transport system with an apparent K_m_ of 103 μM similar to that of the *E. coli* XylE. An ORF located in upstream of the *xylA* gene from a thermophilic *Bacillus* sp. LW2 was cloned and characterized as a xylose transport permease gene (*xylP*) [31]. It was related with the *xylO* gene encoding a putative ATP-binding protein for sugar transport. The *xyl* operon of *Tetragenococcus halophila* used to ferment soy source contains the *xylE* gene similar to that of other Gram-positive or Gram-negative bacteria [32]. XylE of *T. halophila* was likely a xylose:Na^+^ symporter different from other D-xylose transporters using the proton motive force. The *Lactobacillus brevis* *xylABT* operon revealed an arrangement similar to the *B. megaterium* *xylABT* genes, in which XylT protein encoded by the *xylT* gene showed significant similarity (57~60%) to the D-xylose:H^+^ symporters of bacteria [33]. XylT protein had the apparent *K*_m_ value of 215 μM and 6-deoxy-D-glucose inhibited highly the D-xylose transport by XylT. Addition of mannose into culture broth increased the specific growth rate of *L. pentosus* and a deficient mutant of EII^man^ involved in the phosphoenol pyruvate:mannose transferring system could not grow in a minimal medium containing D-xylose only as a carbon source, which suggested that EII^man^ likely transported D-xylose with the facilitated diffusion activity [34]. The mannose transport system revealed the same stimulus on D-xylose uptake of *L. casei* and *L. plantarum* harboring plasmids for the D-xylose-catabolic enzymes. A Gram-positive xylanolytic thermophilic anaerobe, *Thermoanaerobacter ethanolicus* possesses the D-xylose binding protein XylF on the D-xylose utilization operon. Different from other bacterial transport systems, ATP-binding protein or permease of the D-xylose transport system is not located beside the *xylF* gene in *T. ethanolicus*. Kinetic investigation of the xylose transport in *T. ethanolicus* resulted in 1.5 and 200 μM of *K*_m_ values, indicating that there would be another low-affinity xylose uptake system in addition to XylF [35]. A putative *xylGH* operon was identified to encode a putative ATP binding protein (XylG) and a xylose transport permease (XylH) [36]. Specific xylose transport systems and/or its transport kinetic parameters in Gram (+) bacteria were summarized in Table 1. For D-xylose transport, *B. subtilis* 168 was able to grow with xylan (a polymer consisting of xylose) [37], but its growth did not occur in a minimal medium containing D-xylose as a sole carbon source. Such an observation could be explained by absence of the effective D-xylose uptake system in *B. subtilis* [26, 38]. Small amounts of D-xylose for induction of the *xylA* and *xylB* expression were probably transported in an unspecific way. To circumvent insufficient D-xylose uptake, a *B. subtilis* 168 mutant able to take up D-xylose efficiently was isolated [39]. Investigation of the *B. subtilis* *xyl*^+^ mutant clearly exhibited that wild type of *B. subtilis* did not grow on D-xylose as a sole carbon source owing to lack of the specific D-xylose transporter and that a putative xylan transport gene, *xynC* did not encode a D-xylose transporter. Meanwhile, the *B. subtilis* *araE* gene encodes the primary arabinose:H^+^ symport protein (AraE), which contained a typical membrane protein structure of the 12 major hydrophobic regions [26]. Utilization of xylose as a sole carbon source by a *B. subtilis* mutant obtained by random transposon mutagenesis exhibited that AraE is directly or indirectly necessary for transport of galactose and xylose into the *B. subtilis* Gal^+^ strain [40]. A *B. subtilis* can consume xylose moderately in the presence of arabinose, suggesting that AraE expression induced by arabinose addition may be related to xylose transport [41].

### Heterologous Expression of Xylose Transport System

As described before, *B. subtilis* is unable to take up xylose because it doesn’t have a xylose-specific transporter. But, the arabinose:H^+^ symporter, AraE protein is supposed to be able to transport xylose efficiently since arabinose shows a similar molecular structure to xylose, an epimer of arabinose. In order to transport xylose into the cells, the *araE* gene coding for the AraE protein from *B. subtilis* was expressed in engineered *B. subtilis* strains, which has been applied for various biochemicals production [26]. The AraE expression cassette was constructed to contain the xylose-inducible *xylA* promoter, *araE* gene and *fba* terminator, and integrated into the chromosomal *amyE* gene in *B. subtilis* 168. Batch cultures in a defined medium with xylose only or a mixture of xylose and glucose showed that expression of AraE led to fast and complete consumption of initially added xylose and hence a considerable increase in cell growth of the recombinant *B. subtilis* JY123 expressing AraE. Considering the systematic analysis of cell growth, sugar consumption, respiratory quotient and xylulokinase activity, it was certain that AraE protein could transport xylose into *B. subtilis* efficiently [26]. A similar strategy such as constitutive expression of *araE* and deletion of chromosomal *araR* allowed a recombinant *B. subtilis* to produce 430 mg/l fengycin from xylose only [42]. A recombinant *B. subtilis* expressing the *B. subtilis* *araE*, and *E. coli* *xylA*/*xylB* genes under the constitutive P43 promoter showed a typical diauxic growth, complete consumption of glucose and xylose, and increased production of acetoin [43]. Also, the expression of AraE protein provided the enhanced xylose uptake capacity in other bacterium and yeast. The expression of the *Corynebacterium glutamicum* *araE* gene under the control of a constitutive *tac* promoter allowed a 2.9-fold faster growth of recombinant *C. glutamicum* CRX-*araE* using 3.6 g/l xylose only than the parental strain [44]. The *B. subtilis* *araE* gene was expressed in recombinant *Saccharomyces cerevisiae* harboring a foreign xylose reductase, which allowed minimum 40%enhancement in both xylose consumption and xylitol production relative to the case without the *araE* expression [45].

## D-Ribose and Its Biological Production in *B. subtilis*

### D-Ribose and Its Properties in Bio-Health Area

D-Ribose is a five-carbon sugar with C_5_H_10_O_5_ of molecular formula. D-Ribose and its derivatives have been found as a component of most organisms. In the context of commercial applications, D-ribose has long served as the focus for a starting material for the chemical synthesis of riboflavin, which can be used not only for pharmaceuticals but also for animal feed additives, cosmetics and foods. D-Ribose itself has been known to have a cardioprotective effect on adenine nucleotide metabolism in heart muscle of rat [46]. In patients with stable coronary artery disease, administration of D-ribose by mouth for 3 days improved tolerance to myocardial ischemia [47]. Donor treatment and metabolic support with D-ribose during organ preservation might be suitable to prolong the storage time of donor heart due to maintain ATP at a higher level [48]. Supplementation of D-ribose was also tried to increase the skeletal muscle adenine salvage rate during recovery from the intense contraction and subsequently enhanced the muscle performance [49].

### Metabolic Pathway for D-Ribose Biosynthesis

**Fig. 4:**
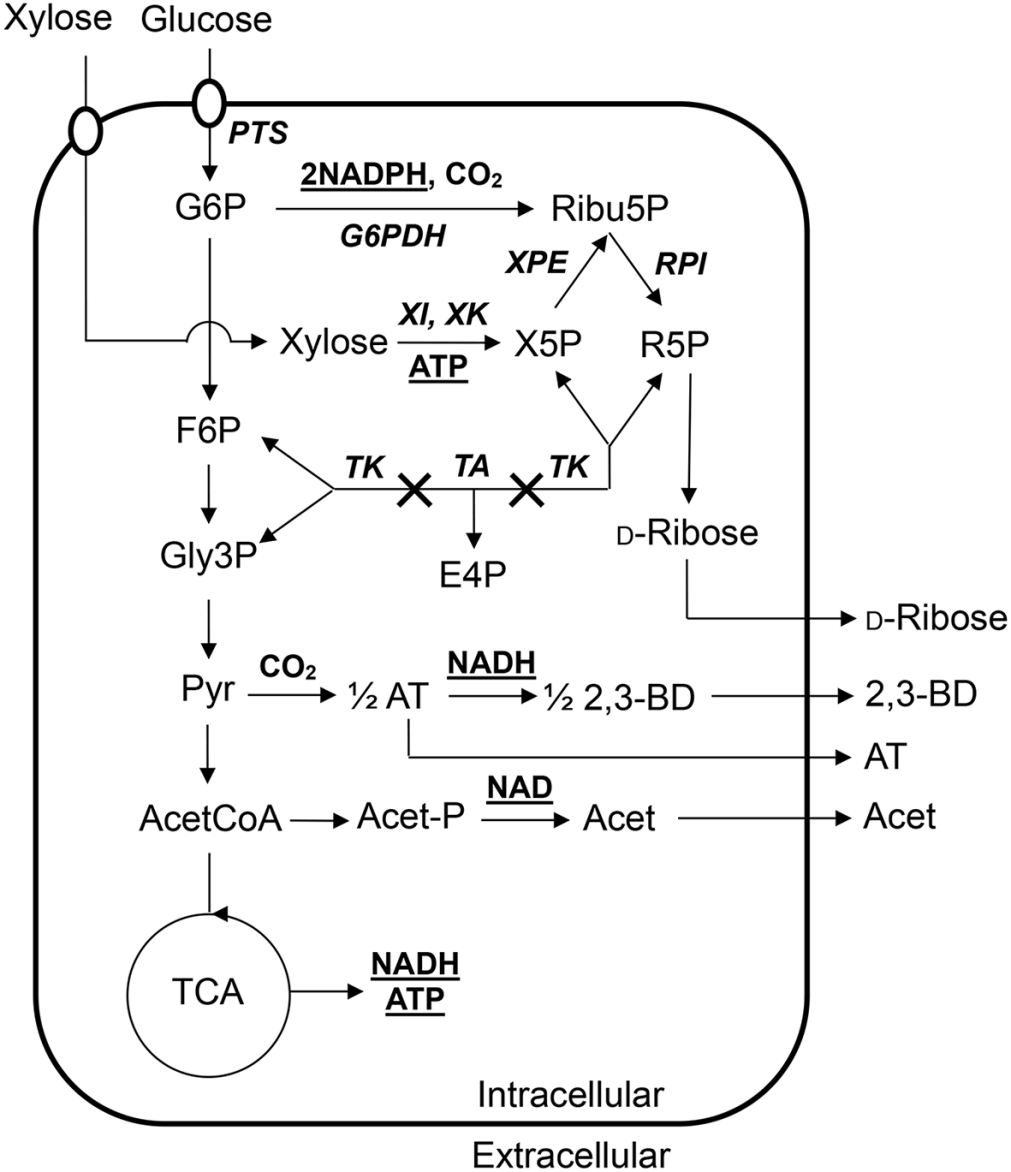
Metabolic pathway for D-ribose biosynthesis from xylose and glucose (modified in a previous report [56]). Bold and underlined compounds showed cofactors produced or consumed in catabolic steps. Bold and italic letters indicated enzymes involved in D-ribose biosynthesis, which abbreviated as follows: G6PDH, glucose-6-phosphate dehydrogenase; XPE, xylulose-5-phosphate epimerase; RPI, ribulose-5-phosphate isomerase; PTS, phosphoenolpyruvate sugar transferring system; TA, transaldolase; TK, transketolase; XI, xylose isomerase; XK, xylulokinase. And other metabolites abbreviated below: G6P, glucose-6-phosphate; F6P, fructose-6-phosphate; Gly3P, glyceraldehyde-3-phosphate; Pyr, pyruvate; AcetCoA, acetyl-CoA; AT, acetoin; 2,3-BD, 2,3-butanediol; Acet-P, acetyl phosphate; Acet, acetic acid; Ribu5P, ribulose-5-phosphate; X5P, xylulose-5- phosphate; R5P, ribose-5-phosphate, E4P, erythrose-4-phosphate. Crosses indicated the disruption of the transketolase gene, leading to blocking the non-oxidative PP pathway. Oval symbols indicated the sugar transport system.

The formation of NADPH and D-ribose-5-phosphate plays a key role in the pentose phosphate (PP) pathway in *B. subtilis* (Fig. 4). NADPH is used in the reductive biosyntheses, whereas D-ribose-5-phosphate is used in the biosynthesis of RNA, DNA, nucleotide coenzymes and ribitol, a component of ribitol teichoic acid in the cell wall of *Bacillus* species. The PP pathway is divided into the oxidative and non-oxidative pathways. In the oxidative PP pathway, glucose-6-phosphate is converted into ribulose-5-phosphate or ribose-5-phosphate, accompanied with two moles NADPH formation and one mole CO_2_ evolution. In the non-oxidative PP pathway, pentose phosphates can enter the glycolysis pathway by way of fructose-6-phosphate and glyceraldehyde-3-phosphate. Transketolase (EC 2.2.1.1) is a metabolic enzyme involved in the non-oxidative PP pathway and creates a reversible link with transaldolase between glycolysis and the PP pathway. If transketolase is deactivated, all of glucose metabolites catalyzed by glucose-6-phosphate dehydrogenase are not directed into glycolysis. In addition, the five carbon sugars such as xylose and arabinose which are metabolized by metabolic enzymes encoded by their own operons may be not utilized as a sole carbon source but accumulate as an intermediate, ribulose-5-phosphate or ribose-5-phosphate in the PP pathway because of blocking the non-oxidative PP pathway. Microbial D-ribose production is carried out by dephosphorylation of cumulative ribose-5-phosphate caused by transketolase disruption.

### Production of D-Ribose from Xylose and Other Sugars

Various methods have been investigated and established for the large-scale production of D-ribose. D-Ribose was obtained by the chemical hydrolysis or the enzymatic hydrolysis of yeast ribonucleic acid (RNA), of which a component is D-ribose. Bacterial nucleosidases and some chemical catalysts were used to hydrolyze 5-amino-4-imidazole-carboxamide riboside for production of D-ribose [50]. Chemical methods for D-ribose synthesis were established using cheap materials such as arabinose, glucose and gluconate. A continuous process of D-arabinose epimerization was developed in a reaction tube with the molybdenum (VI) compound [51] and the acid hydrolysis of a 1,5-di-*O*-alkyl derivative of D-xylofuranose [52] was also devised to synthesize D-ribose.

Microbial production of D-ribose has been developed mostly using bacteria including *Bacillus* species. By several random mutations and screening, some mutants lacking the metabolic enzymes engaged in the pentose phosphate pathway were able to produce D-ribose in culture medium. Interestingly, these did not show any transketolase activity. Most *B. subtilis* accumulating D-ribose were selected and characterized as transketolase mutants [53]. Efforts to develop the *tkt* (encoding transketolase) mutants of bacterial strains such as *B. subtilis*, *B. pumilus*, *Brevibacterium thiogenitalis*, *B. ammoniagenes*, *Arthrobacter globiformis*, *Aerobacter aerogenes* and *Micrococcus denitrificans* revealed that only *Bacillus* species could accumulate D-ribose [1, 53, 54].

Many research efforts have been made to establish the fermentation processes for the mass production of D-ribose from xylose and other sugars by the transketolase mutants of *B. subtilis* as summarized in Table 2. When a D-ribose producing *B. subtilis* mutant (ATCC 21951) was grown on glucose plus a second substrate (D-xylose, D-gluconate, L-arabinose and xylitol), catabolite repression on utilization of the second substrate was not observed, and hence simultaneous xylose consumption along with fast utilization of glucose led to the production of 60 g/l D-ribose with 0.55 g/l-h productivity [55]. A transketolase-deficient *B. subtilis* mutant strain of JY1 was identified to lose the glucose-specific enzyme II of the phosphoenolpyruvate transferase system, and simultaneously converted both xylose and sucrose to D-ribose with a yield of 0.43 g/g sugars [1]. A chemical mutant of *B. subtilis* SPK1 was constructed from the transketolase-deficient *B. subtilis* JY1 and produced 23.0 g/l D-ribose from 20 g/l xylose and 20 g/l glucose, which was 1.7 times higher than those for the parent strain (JY1) [56]. A fed-batch culture strategy with the feeding of the 200 g/l xylose and 50 g/l glucose solution at the late-exponential growth phase increased the final concentration of D-ribose to 46.6 g/l with 1.16 g/l-h productivity [56]. And composition of xylose and glucose in the culture medium and air supply strategy were also important for D-ribose production, in which a composition of 40 g/l xylose and 41 g/l glucose, and aeration with 600 rpm and 1 vvm were optimal for cell growth, sugar consumption and D-ribose production [2].

Except for xylose, the most popular carbon source of glucose has been used as the main carbon source for D-ribose production [53]. A highly aerated batch fermentation of *B. subtilis* ATCC 21951 yielded 40 g/l D-ribose from 200 g/l glucose. By using 100 g/l glucose and 50 g/l gluconic acid (a metabolite in the oxidative pentose phosphate pathway), 45 g/l D-ribose was achieved in the same batch culture [57]. Supplement of nitrogen sources (casamino acid, corn steep liquor) and an intermediate (citrate) gave the positive effects on D-ribose production from glucose in batch cultures of *B. subtilis* IFO 13322 [58], EC2 [59] and UJS0717 [60]. A fed-batch cultivation of *B. subtilis* CGMCC 3720 using 60 g/l of initial glucose was developed by co-feeding 2.2 g/l-h glucose and 0.036 g/l-h citrate, in which formation of organic acid byproducts was limited, and hence D-ribose production was enhanced to 113.4 g/l [61].

Other technologies of genetic engineering and enzymatic conversion have been also applied for D-ribose production. By genetic engineering, the chromosomal *tkt* gene was disrupted directly in the type-strain of *B. subtilis* 168 and its resulting recombinant *B. subtilis* strains were characterized by fermentation technology. A transketolase-deleted recombinant *B. subtilis* JY200 was cultured at a fed-batch strategy of both feeding of 400 g/l glucose and maintaining of 10 g/l xylose in the culture broth, in which 10.1 g/l D-ribose with 0.24 g/g yield were obtained [54]. Also, *B. subtilis* SFR-3A strain with the full disruption of the *tkt* gene was able to covert 110 g/l glucose to 27.6 D-ribose with 0.51 g/l-h productivity [62]. A recombinant *Escherichia coli* SGK015 also produced 3.75 g/l D-ribose from glucose and xylose, in which the chromosomal *tktA* gene encoding transketolase and the *ptsG* gene encoding the glucose-specific IICB component were disrupted by genetic engineering [63]. Meanwhile, enzymatic routes from D-xylose to D-ribose were developed. A izumoring route was designed with a glucose isomerase from *Acidothermus cellulolyticus* 11B (AcceGI), a D-allulose 3-epimerase from *Clostridium cellulolyticum* H10 (CcDPEase) and a L-rhamnose isomerase from *B. subtilis* 168 (BsLRhI), and hence 9.55 g/l D-ribose with 0.3 g/l-h productivity from about 30 g/l xylose was produced by stepwise addition of xylose [64]. Another enzymatic method was developed to highly increase the conversion yield of xylose to D-ribose. By adoption of selective phosphorylation and dephosphorylation process using five enzymes, 93.5 % mol/mol of D-ribose yield from xylose was achieved [65].

## Future Perspectives

Xylose metabolim in *B. subtilis* is regulated by CCR in the presence of glucose and xylose plays a role in transcriptional inducer of the *xylA* and *xylB* gene expression. The strong transcriptional capacity of the xylose promoter is suggested to be used in the foreign gene expression in recombinant *B. subtilis*. However, *B. subtilis* does not possess a xylose-specific transporter, so that the arabinose transporter encoded by the *araE* gene should be expressed for efficient xylose uptake as described in the simulatenous engineering of the xylose transporter and the *araE* overexpression in *B. subtilis* [26]. As described in the D-ribose production, *B. subtilis* could be applicable for production of other valuable biomaterials because it is a GRAS microorganism with various records for commercial choice and many tools for micrbial and fermentation engineering.

## Figures and Tables

**Table 1:** Xylose transport systems in Gram-positive bacteria.

Organism	Transport protein	Driving force	Homology^1)^ (%)	K_m_ (mM)	Reference
*Bacillus subtilis*	AraE	H^+^			[26]
*Bacillus megaterium*	XylT	H^+^	54	0.10	[22]
*Bacillus* sp. LW2	XylP	ATP	47.7		[31]
*Lactobacillus brevis*	XylT	H^+^	57	0.22	[33]
*Lactobacillus pentosus* casei plantarum	EII^man^ ^2)^	F.D.^3)^		8.5	[34]
*Tetragenococcus halophila*	XylE	Na^+^	51		[32]
*Thermoanaerobacter ethanolicus*	XylF	H^+^		1.5 × 10^-3^	[35]
	XylGH	ATP		0.2	[36]

^1)^Homology percentages were obtained by the comparison with the *xylE* gene of *E. coli*.

^2)^EII^man^ indicated the protein II complex in the phosphoenolpyruvate:mannose transferring system.

^3)^F.D. abbreviated facilitated diffusion.

**Table 2:** D-ribose production in *Bacillus subtilis* strains without transketolase.

*B. subtilis*	Culture type	D-ribose			Reference
		Concentration (g/l)	Yield (g/g sugar)	Productivity (g/l-h)	
ATCC 21951	Batch (1 L), 100 g/l glucose + 100 g/l xylose	60.0	0.30	0.55	[55]
JY1	Batch (1 L), 15.2 g/l xylose + 8.6 g/l sucrose, 37°C, 600 rpm, pH7.0, 1 vvm	12.0	0.43	0.30	[1]
SPK1	Fed-batch (1 L), feeding 200 g/l xylose + 50 g/l glucose at the late exponential growth phase	46.6	0.43	1.16	[56]
SPK1	Batch (1 L), 40 g/l xylose + 41 g/l glucose, 37°C, 600 rpm, 1 vvm	35.6	0.67	0.81	[2]
ATCC 21951	Batch (1 L), 200 g/l glucose, 37°C, pH7.0, 3 vvm	40.0	0.40	0.26	[57]
IFO13322	Batch (1 L), 200 g/l glucose + 15 g/l casamino acid	53.1	0.43	0.55	[58]
EC2	Batch (15 L), 180 g/l glucose + 0.3 g/l citrate, 37°C, pH 6.0, 400 rpm, 1 vvm	83.4	0.49	1.99	[59]
UJS0717	Batch, 157 g/l glucose from corn starch hydrolysate, 21 g/l corn steep liquor, 36°C, pH7.0	62.1	0.40	0.86	[60]
CGMCC 3720	Fed-batch, co-feeding of 2.22 g/l-h glucose and 0.036 g/l-h sodium citrate	113.4	-	1.58	[61]
JY200	Fed-batch (1 L), feeding 400 g/l glucose, maintaining 10 g/l xylose in culture broth,	10.1	0.24	0.29	[54]
SFR-3A	Batch, 110 g/l glucose, 37°C, pH6.5, 15% dissolved oxygen	27.6	0.25	0.51	[62]

Most the *Bacillus* strains without transketolase activity were selected or constructed by random mutagenesis except for JY200 and SFR-3A, in which the chromosomal *tkt* gene was genetically disrupted in the host strain of *B. subtilis* 168.
